# Improving quantitative writing one sentence at a time

**DOI:** 10.1371/journal.pone.0203109

**Published:** 2018-09-12

**Authors:** Tracy Ruscetti, Katherine Krueger, Christelle Sabatier

**Affiliations:** Biology Department, Santa Clara University, Santa Clara, California, United States of America; Fordham University, UNITED STATES

## Abstract

Scientific writing, particularly quantitative writing, is difficult to master. To help undergraduate students write more clearly about data, we sought to deconstruct writing into discrete, specific elements. We focused on statements typically used to describe data found in the results sections of research articles (quantitative comparative statements, QC). In this paper, we define the essential components of a QC statement and the rules that govern those components. Clearly defined rules allowed us to quantify writing quality of QC statements (4C scoring). Using 4C scoring, we measured student writing gains in a post-test at the end of the term compared to a pre-test (37% improvement). In addition to overall score, 4C scoring provided insight into common writing mistakes by measuring presence/absence of each essential component. Student writing quality in lab reports improved when they practiced writing isolated QC statements. Although we observed a significant increase in writing quality in lab reports describing a simple experiment, we noted a decrease in writing quality when the complexity of the experimental system increased. Our data suggest a negative correlation of writing quality with complexity. We discuss how our data aligns with existing cognitive theories of writing and how science instructors might improve the scientific writing of their students.

## Introduction

Written communication of data is at the core of scholarly discourse among scientists and is an important learning goal for science students in undergraduate education [1]. For scientists, the currency of scientific dialogue is the research article, which presents essential information required to convince an audience that data are compelling, findings are relevant, and interpretations are valid [2, 3]. Writing lab reports that contain the elements of a research article is a widely used method to help students develop critical thinking and quantitative reasoning skills. In our introductory, lab-intensive Cell and Molecular Biology course, we focus on helping students develop the “results” section of their lab report. Students integrate tables, graphs, and text to present and interpret data they have generated in the laboratory. In the text portion, students cannot simply restate previously learned information (“knowledge telling;” [4, 5]) or narrate through the data presented visually. Rather, students must mimic the actions of professional researchers by transforming data into knowledge and structuring their arguments to support specific claims/conclusions. This type of inquiry-based writing encourages active participation in the scientific process, enhancing engagement and learning [6, 7].

While science instructors recognize the importance of writing in their courses, many do not provide explicit writing instruction [8]. Instructors may fear that teaching writing skills diverts time from teaching required science concepts, expect that writing is covered in composition courses, or lack the tools and resources to teach writing [8, 9, 10]. We wanted to support writing in our course without diverting focus from the conceptual and discipline-specific content of the course. We examined available writing resources (e.g., books, websites) and found substantial resources regarding the macro structure of the report (e.g., describing the sections and broad organization of the lab reports, [11, 12]. We also found resources for sentence level support related to emphasis and voice [13]. However, these resources do not give students explicit guidance as to how to write about quantitative information. Thus, it is not surprising that many students struggle to both construct appropriate quantitative evidence statements and express them in writing [14].

There are, however, a few important resources that explore the structure of writing about quantitative information. Each describe comparisons as a primary mode of providing quantitative evidence, (e.g., *The lifespan of cells grown in the presence of drug is 25% shorter than the lifespan of control cells*.). In her book about writing about numbers, Miller discusses “quantitative comparisons” as a fundamental skill in quantitative writing [15]. Jessica Polito states that many disciplines use comparisons as the basis of quantitative evidence statements that support conclusions [14], and Grawe uses the presence of a comparison as a measure of sophisticated quantitative writing [16]. We focused on these types of comparative evidence statements and called them Quantitative Comparative statements (QC). We found this type of statement was commonly used to describe data in the scientific literature, and we decided to emphasize the correct construction of these statements in student writing.

We analyzed over a thousand QC statements from student and professional scientific writing to discover the critical elements of a QC statement and the rules that govern those elements. We found that a QC statement needs to have a comparison, a quantitative relational phrase, and at least one contextual element. These essential elements of the QC statement can be thought of as sentence-level syntax. We then developed a metric to measure writing syntax of the QC statement and by proxy, quantitative writing quality. We examined the effectiveness of different approaches to support writing in a course setting and show that practice writing QC statements with feedback can improve student writing. We also investigated how the circumstances of the writing assignment can change the quality of quantitative writing. Together, these data provide insight into how we might improve undergraduate science writing instruction and the clarity of scientific writing.

## Methods and materials

### Student population and course structure

We collected data at Santa Clara University (SCU), a private liberal arts university that is a primarily undergraduate institution. Participants were recruited from BIOL25 –Investigations in Cell and Molecular Biology, a lower-division biology course. Prerequisites include a quarter of introductory physiology, a year (3 quarters) of general chemistry and one quarter of organic chemistry. BIOL25 consists of three interactive lecture periods (65 minutes) and one laboratory period (165 minutes) per week. The lecture periods focus on preparing for the laboratory experience, analysis, interpretation, and presentation of data. Laboratory sessions focus on data collection, data analysis and peer feedback activities. During the 10-week quarter, two experimental modules (Enzyme Kinetics and Transcription Regulation) culminate in a lab report. Students organize and communicate their analyzed data in tables and graphs and communicate their conclusions and reasoning in written form. We provide a detailed rubric for the lab reports and a set of explicit instructions for each lab report (S2 Fig). In addition, students participate in peer feedback activities with an opportunity to revise prior to submission.

The basic structure of the course was unchanged between 2014 and 2016. The students were distributed among two lecture sections taught by the same instructors and 13 laboratory sections led by 5 different instructors. All students included in this study signed an informed consent form (213 of 214). This study was reviewed and approved by the Santa Clara University Institutional Review Board (project #15-09-700).

### Instructional support

#### General writing feedback (2014–2016)

In all iterations of the course discussed in this article, students received general writing feedback after each lab report. In each lab report, students wrote paragraphs in response to prompting questions regarding the data. Writing feedback was holistic and included phrases such as “not quantitative”, or “inappropriate comparison,” but was not specific to any type of sentence.

#### Calculation support (2015–2016)

In 2015 and 2016, students were explicitly introduced to strategies for quantifying relational differences between data points such as percent difference and fold change. Students were given opportunities to practice calculating these values during in class activities prior to writing their lab reports. We stressed that phrases such as more than, drastically higher, and vanishingly small were not quantitative.

#### Explicit QC statement writing support (2016)

In 2016, we introduced and practiced using quantitative comparative statement as the means to communicate quantitative results. In class, we discussed including an explicit comparison of two conditions and the quantitative relationship between them. Before each lab report, we asked students to write quantitative comparative statements related to the data. We provided formative feedback on the accuracy of the statement and general feedback such as, “not quantitative”, or “inappropriate comparison”. Students in this study were never exposed to the concept of 4C annotation or scoring. We used the scoring strategy exclusively to measure their writing progress.

### Identification of quantitative comparative statements (QC)

Quantitative comparative statements are a subset of evidence statements. In native writing (scientific articles or student lab reports), we identified QC statements by the presence of 1) a relational preposition (between, among, etc.), or 2) prepositional phrase ("compared to", "faster/slower than", etc.), 3) a statistical reference (p value), or 4) the presence of quantified change (3 fold, 10% different).

### Syntactic elements of QC statements

We examined a corpus of over 1000 QC statements to identify and characterize the essential elements of a QC statement and the rules that govern those elements. Quantitative comparative statements generally take the form of “*The activity of the enzyme is 30% higher in condition X compared to condition Y*”. We identified three critical elements of the quantitative comparative statement: the things being compared (Comparison, *condition X* and *condition Y*), the quantitative relationship between those conditions (Calculation, *30% higher*), and the measurement that gave rise to the compared values (Context, *enzyme activity*). Finally, all three elements must be in the same sentence with no redundancy or contradiction (Clarity). These rules are collectively called “4C”.

### Syntactic rules for quantitative comparative statements

The Calculation must quantify the relationship between the two compared elements and include both magnitude and direction. Fold change or percent difference are common methods of describing quantitative relationships [15]. Using absolute or raw values are not sufficient to describe the relationship between the compared elements and are not sufficient. If there is no significant difference between the compared elements, then statistical data must be cited. *Context* provides additional information about the measurement from which the quantitative comparison was derived, such as growth rate, enzyme activity, etc., or the time at which the comparison was made. The context should be the same for both of the compared elements. Comparisons are usually between like elements (e.g. time vs. time, condition vs. condition) and there should be two and only two in a single sentence. Both compared elements must be explicitly stated so that the reader is not guessing the intended comparison of the writer. A QC statement has Clarity when all three elements are present and in the same sentence. We consider a statement to be “unclear” if it contains inconsistencies or redundancies.

### Annotation and scoring of QC statements

**Fig 1:**
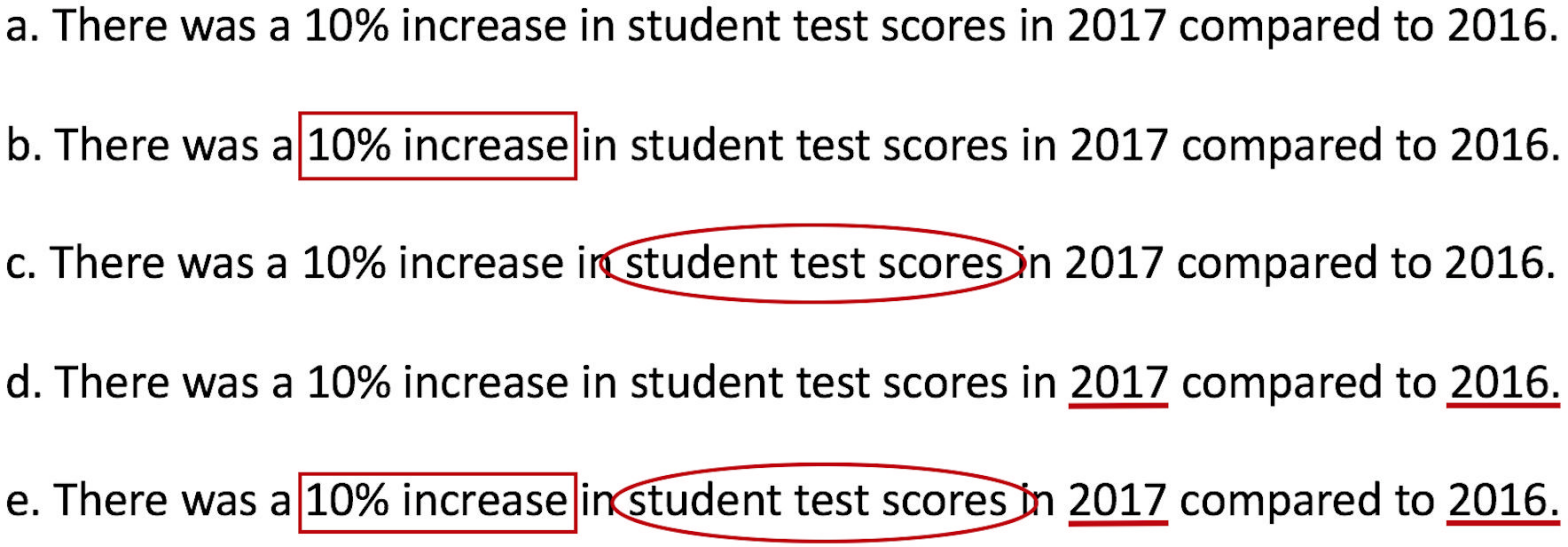
4C annotation of a quantitative comparative statement. (A) Original quantitative comparative statement. (B) Identify and box the relational phrase with both magnitude and direction. (C) Circle what the relational phrase refers to (context). (D) Underline the comparison. (E) Fully 4C annotated quantitative comparative statement.

We use “annotation” to describe the visual marking of the critical elements of the quantitative comparative statement. We use “scoring” to mean the assignment of a score to a quantitative comparative statement. 4C annotation and 4C scoring do not reflect whether the statement or any of its components are correct, but rather they highlight the syntactic structure of the quantitative comparative statement (Fig 1).

#### Annotation process

We scanned the results sections of published primary journal articles or student lab reports for relational phrases such as faster than, increased, more than, lower than, etc., and **drew a box around the relational phrase**, or ***calculation*** (Fig 1B). If the calculation is an absolute value, a raw value, refers to no particular value, or is missing the magnitude or direction, we would strike through the box. *Context*. Once the relational phrase, or calculation, was identified, we **drew a circle around the information, or context**, referred to by the relational phrase (Fig 1C). *Comparison*. The relational phrase and the context helped us identify the comparison and we **underlined the compared elements** (Fig 1D).

#### 4C scoring strategy

To score an annotated statement, a “1” or a “0” is given to each of the three critical components of the quantitative comparative statement. If all the elements are present in a single sentence, there are no redundancies or inconsistencies, a fourth “1” is awarded for clarity. We call this annotation and scoring strategy “4C” to reflect each of the three critical components and the overall clarity of the statement (Table 1).

**Table 1:** Sample statements of common errors defined by 4C scoring.

		4C scoring				
Annotated statement	Calc	Cont	Comp	Clarity	4C Score	Issues
In 2016, we measured a 75% increase in student lab report scores compared to the same assignment in 2015.	**1**	**1**	**1**	**1**	**4**	All elements are present and proximate
The lactose condition shows a 5-fold increase in β-Galactosidase activity at t = 60.	**1**	**1**	**0**	**0**	**2**	Missing comparative element
In the 5 mM glucose condition, the Miller Units decrease 8-fold from t = 0 to t = 120 (Table 2, t4, p<0.001, Vassarstats) and in the 0.25 mM glucose condition the Miller Units decrease 5-fold from t = 0 to t = 120 (Table 2, t4, p<0.0002, Vassarstats).	**1**	**1**	**1**	**0**	**3**	Multiple comparisons in a single sentence decreases clarity of both. Each comparison is scored separately
	**1**	**1**	**1**	**0**	**3**	
Miller units of the 0.25mM Glucose condition at T = 120 mins decreased 42% as compared to the minimal media and in the 5mM Glucose condition decreased 61%	**1**	**1**	**1**	**0**	**3**	Multiple comparisons in a single sentence. Each comparison is scored separately
	**1**	**1**	**0**	**0**	**2**	
The doubling time at t = 60 was 136, which decreased from t = 45.	**0**	**1**	**1**	**0**	**2**	Relational phrase lacks magnitude
Additionally, looking at the graph of cell concentrations, there is no major difference in cell concentrations between the mixed sugar and 0.25 mM Glucose conditions.	**0**	**1**	**1**	**0**	**2**	Needs statistical support.
The percent difference in Km between glucose and no sugar is only 19.8%.	**0**	**1**	**1**	**0**	**2**	Lacking direction of change.
Enzyme activity in the mixed sugar condition increased 3-fold, from 143 ± 7 miller units at t = 0 to almost 400 miller units at t = 120.	**1**	**1**	**1**	**0**	**3**	Redundant information. No need to restate data from tables.
From T = 0 to T = 120, there’s a 7-fold induction between those two time points.	**1**	**0**	**1**	**0**	**2**	Missing context
In the assays with 25 mM galactose, the average Km and Vmax were 37% and 66% higher than in assays with no sugars, respectively.	**1**	**1**	**1**	**0**	**3**	More than one comparison using complex structure.

### Student writing samples

#### Pre-test/Post-test

In 2016, student writing was assessed using identical pre- and post-tests. The pre-test was administered on the first day of class prior to any writing support. The post-test was administered as part of the final exam. The pre/post assessment consisted of a graph and data table (S1 Fig). The prompts asked the students to analyze the data to answer a specific question related to the data and to use quantitative comparative statements.

#### Student sampling for lab report analysis

For the lab reports in 2016, we sampled 40 students from a stratified student population (based on overall grade in the course) and 4C scored all of their quantitative comparative statements in each lab report. On average, students wrote 5–6 quantitative comparative statements per results section for a total of over 200 4C scored statements for each lab report. We scored over 100 statements from 17–20 lab reports in 2014 and 2015.

### Complexity index

We based complexity on the number of values (data points) students would have to parse to develop a QC statement. The complexity of a given experiment is in part determined by number of conditions tested in an experiment and the different types of measurements used. For example, in lab report #1 (Enzyme Kinetics) students consider 3 experimental conditions (control and two separate variables) and 2 measurements (K_m_ and V_max_). Thus we calculated a complexity index of 6 (3 conditions x 2 measurements) for lab report #1. In this measure of complexity index, we assumed that all parameters contributed equally to the complexity of the experiment, and that all parameters were equally likely to be considered by students as they developed their written conclusions. However, by designing specific writing prompts, we could guide students to examine a smaller subset of data points and reduce complexity of the situation. In lab report #1 for example, we can prompt students to consider only the effect of the treatment on a single variable such that they only consider 2 conditions (the control and the single experimental variable described in the prompt) and 2 measurements. Now, students are focused on a subset of data and the complexity of the situation could be considered “4”.

## Results

### Quantitative comparative statements are universally used to describe data

**Fig 2:**
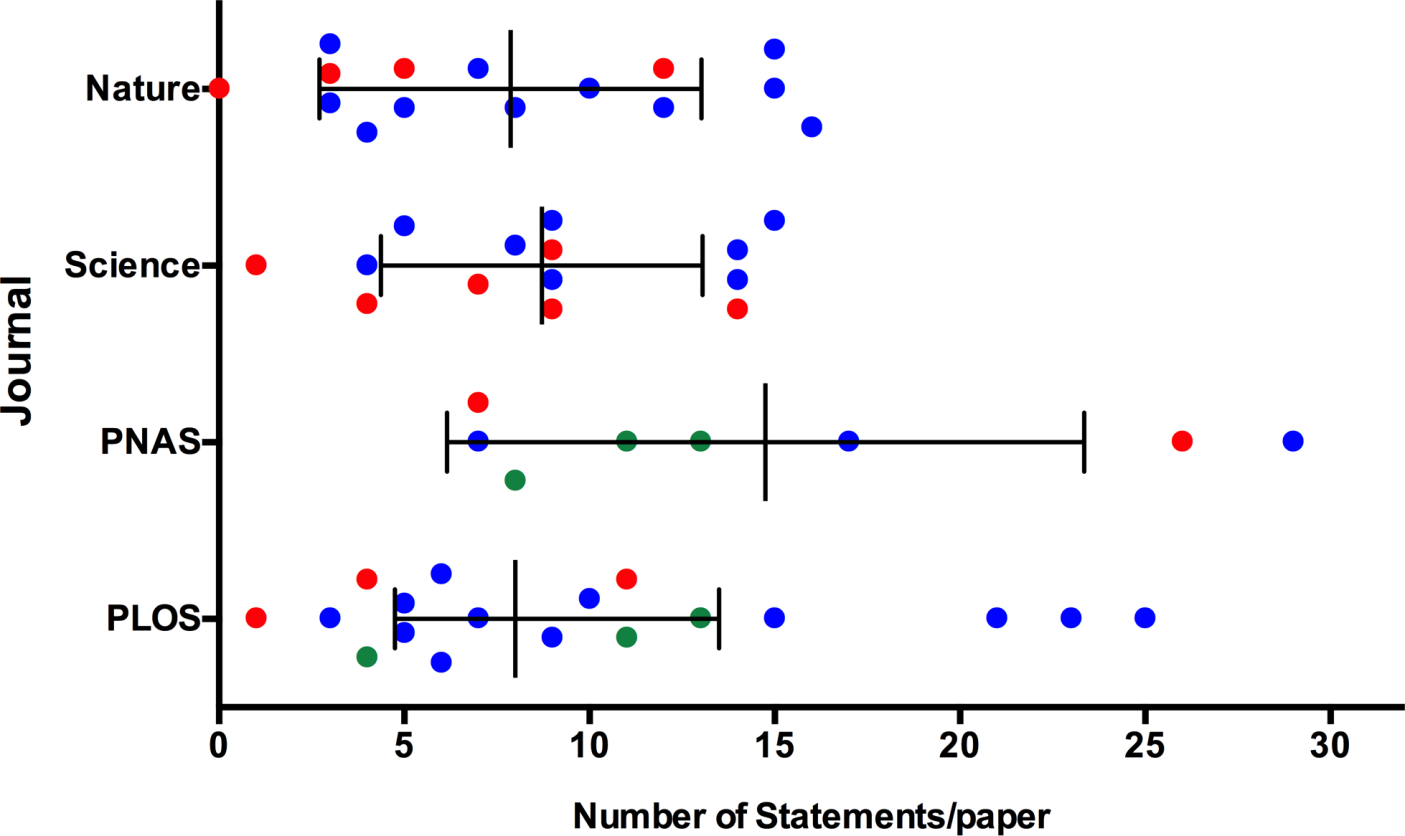
Quantitative comparative statements from results section of published research articles in major pan-discipline journals. The mean (middle vertical line) ± SD are shown. Physical science papers are denoted in red, Biological sciences are in blue, and Social sciences are in green.

Having decided to focus on QC statements in student writing, we first wanted to quantify their occurrence in professional writing. We examined the results sections in all the research articles from three issues of pan-scientific journals: Science, Nature, PLOS-One, and PNAS. We identified an average of 7–15 QC statements in each research article, with no significant difference in the mean number of QC statements among the different journals (Fig 2, ANOVA, p = 0.194). There was also no difference of the number of QC statements among the different disciplines (Kruskal-Wallis, p = 0.302). Out of the 60 articles examined, we found only one article that did not have a single QC statement to describe the data (Fig 2, Nature). These data suggest that QC statements are used in professional forms of quantitative writing to describe data in many different disciplines.

### 4C scoring used to measure quantitative writing

**Fig 3:**
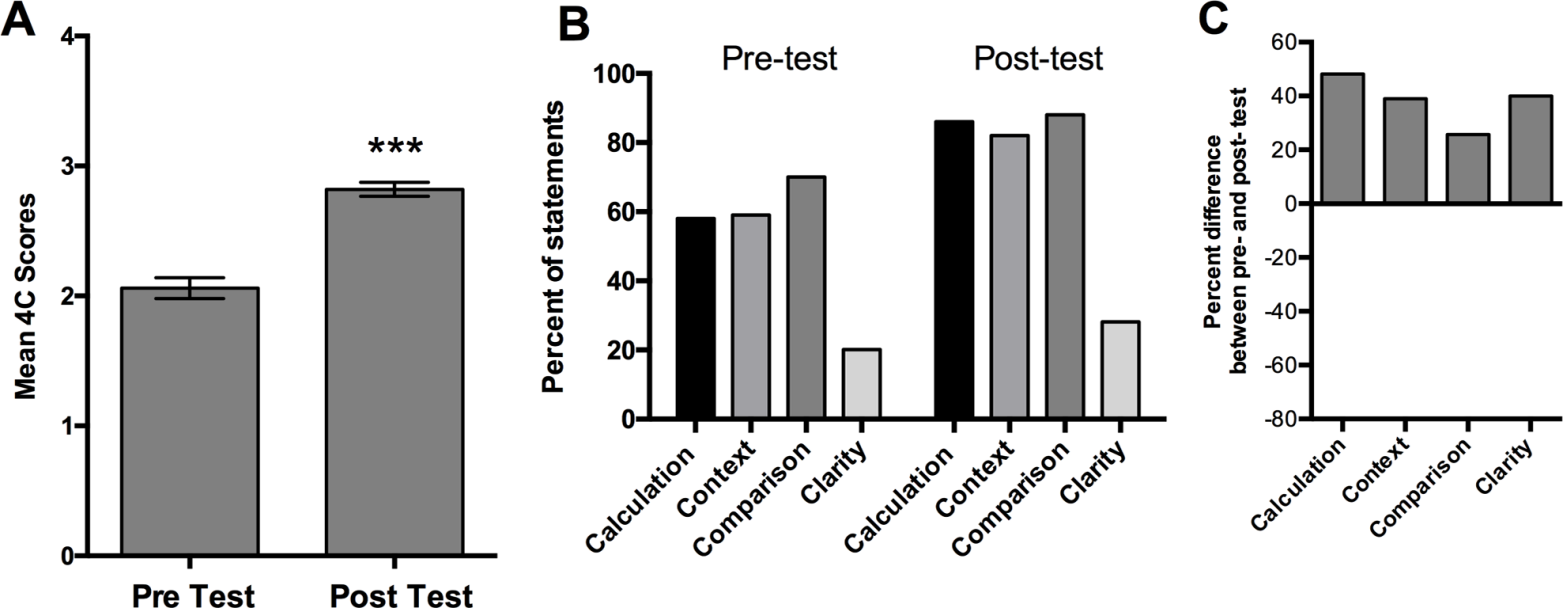
Using 4C scoring to measure quantitative writing before and after instruction. **(**A) Mean 4C scores of quantitative comparative statements on an identical pre- and post- test. (B) Percent of statements that contain each of the essential components of a QC statement. (C) Percent difference between the pre-test and post-test broken down by essential components of QC statements. (***t-test, p < 0.001) Error bars in A represent Standard Error of the Mean (SEM).

In 2016, students practiced writing QC statements related to their data and we provided feedback (see Methods). We measured the effectiveness of the focused writing practice using 4C scoring of QC statements from a pre- and post-test (see Methods and Table 1). We observed a 37% increase in student 4C scores on the post-test assessment compared to the pre-test (p < 0.001, Fig 3A). In addition, we used 4C scoring to interrogate the impact of the writing intervention on each of the required components of the QC statement (Fig 3B). We observed improvements in each of the components of QC statements (Fig 3C). In the post-test, over 80% of students included a calculation (magnitude and direction), referred explicitly to both items being compared, and referenced the measurement context for their comparison. Only 25% of students produced completely clear statements, meaning that they were not missing any elements, and did not contain redundant or contradictory phrases. Despite the low post-test clarity score, we observed a 40% improvement in students writing completely clear statements in the post-test compared to the pre-test score (Fig 3C).

**Fig 4:**
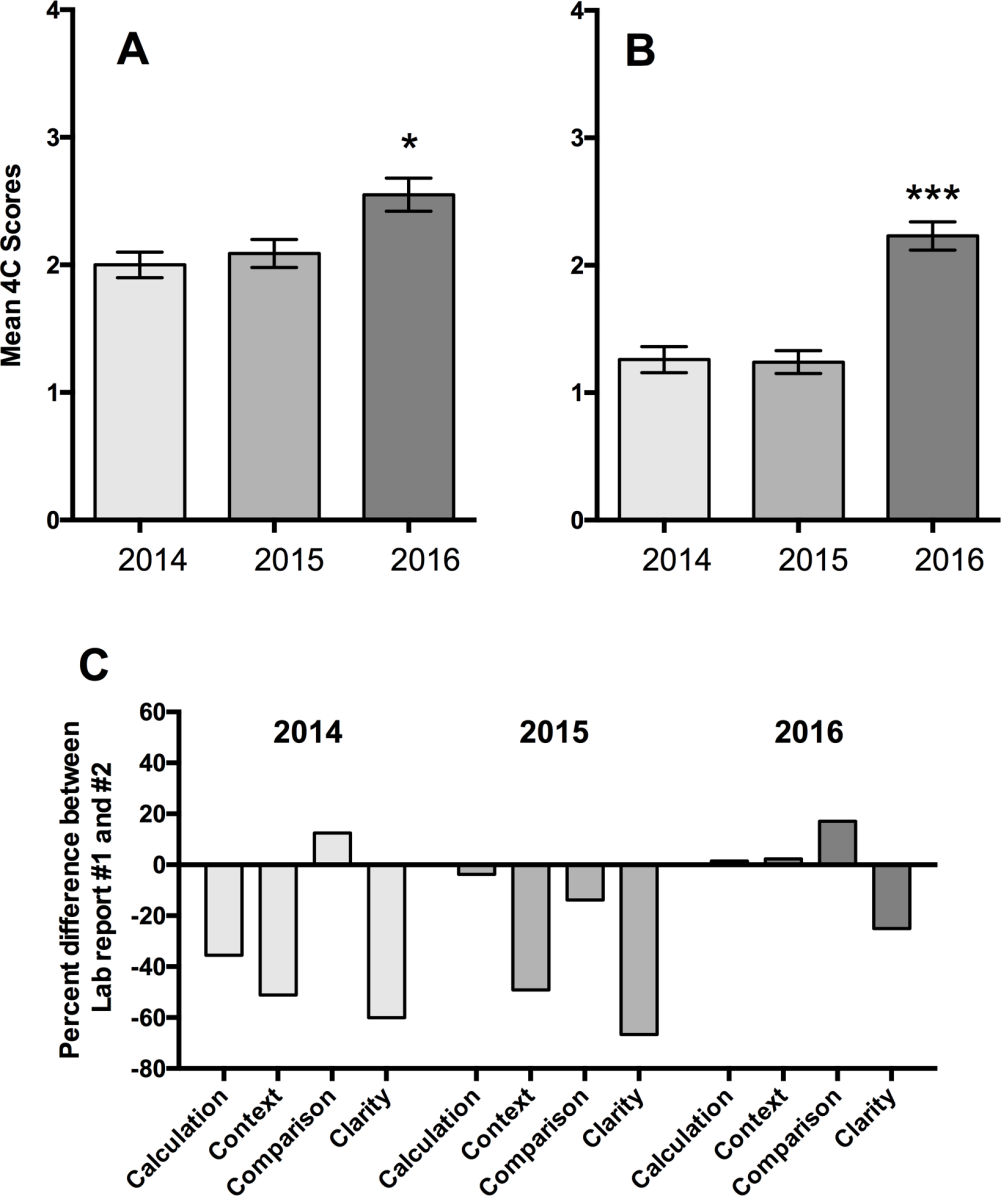
Analysis of native writing samples (lab reports). (A) Mean 4C scores of QC statements from lab reports (enzyme kinetics). (B) Mean 4C scores of QC statements from second lab reports (transcriptional regulation). (C) Percent difference between the two lab reports within a given year, broken down by essential components (*p < 0.05, ***p < 0.001) Error bars in A and B represent SEM.

We next asked if we could measure student learning gains in quantitative writing within the context of a lab report. Students write 2 lab reports per term and we provided varying forms of writing feedback over several iterations of the course (see Methods). We scored QC statements in two lab reports from 2014 (general writing feedback only), 2015 (general writing feedback and calculation support) and 2016 (general writing feedback, calculation support, and sentence-level writing practice) (Fig 4A). There was no appreciable impact on writing quality when we added calculation support to general feedback in 2015 compared to feedback alone in 2014 (t test, p = 0.55, Fig 4A). However, the addition of sentence-level QC writing support in 2016 resulted in a 22% increase in student mean 4C scores on lab report #1 compared to the same report in 2015 (Fig 4A, t test, p < 0.05). We noticed the same trends in lab report #2 (Fig 4B): general writing feedback and calculation support did not improve scores as compared to general feedback alone (t test, p = 0.88). However, we observed an 80% increase in 4C scores on lab report #2 when we provided sentence-level writing practice compared to feedback alone (Fig 4B, t test, p < 0.001). The mean 4C scores in each year for each assessment, as well as the forms of writing support employed, are summarized in Table 2. Overall, these data suggest that sentence-level writing practice with feedback is important in helping students improve the syntax of quantitative writing.

**Table 2:** Mean 4C scores (± SEM) of quantitative comparative statements.

Term	Pre-test	Writing Support	Lab report 1	Lab report 2	Post test
2014	NA*	General feedback	2.00 ± 0.01 (100, 24)	1.26 ± 0.10 (100, 23)	NA
2015	NA	Feedback and Calculation	2.09 ± 0.11 (100, 27)	1.24 ± 0.09 (100, 21)	NA
2016	2.06 ± 0.08 (214, 214)**	Feedback, Calculation and Practice	2.55 ± 0.13 (213, 40)	2.23 ± 0.11 (231, 40)	2.82 ± 0.05 (212, 212)

*the pre- and post- assessments were not administered in 2014 or 2015.

**(number of statements, number of students from which statements were collected)

We were surprised to find that although the trends in the data were similar between the two lab reports, the mean 4C scores of QC statements in lab report #2 were 40% lower than in lab report #1 in both 2014 and 2015 (t test, p < 0.0001, Fig 4A versus 4B). We predicted that writing skills would either improve with focused practice, or not change over the course of the quarter. To understand which components of the quantitative comparative statement were differentially impacted in the two lab reports, we calculated the relative frequency with which each component was included in a QC statement. Then, we calculated the difference of those frequencies between the first and second lab report for each year (Fig 4C). A column below the x-axis indicates that students made particular mistakes more often in lab report #2 (Fig 4C). In 2014, students were able to make comparisons equally well between both lab reports, but students struggled to include a quantitative difference or provide context in their evidence statements (Fig 4C). In 2015, in addition to general writing feedback, we also provided instructional support to calculate relative differences. We noted that students were able to incorporate both comparisons and calculations into their QC statements in both reports. However, they often omitted the context (Fig 4C). The frequency of mistakes made by students is significantly different between lab report #1 and lab report #2 (Chi squared, p < 0.001). These data suggest that feedback alone is not sufficient to improve quantitative writing. In 2016, we provided targeted practice at the sentence level and observed no significant difference in mean 4C scores between the two lab reports (Fig 4B, t test, p = 0.0596), suggesting that the writing skills of students did not decrease from one lab report to the next. Additionally, students included the four elements of the QC statement equally well between the two lab reports (Chi squared, p = 0.6530, Fig 4C, 2016). Thus, when students receive targeted, sentence-level writing practice, their ability to write QC statements improves.

### Quantitative writing quality is negatively impacted by complexity

We were perplexed as to why quantitative writing syntax (as measured by mean 4C scores) declined in lab report #2 compared to lab report #1 in both 2014 and 2015 (Fig 4A and 4B). Because we view the essential components of QC statements as analogous to syntactic rules that govern writing of QC statements, we can apply principles and theories that govern writing skills *writ* large. Research from writing in English Composition shows that writing ability, as measured by sentence level syntax, deteriorates when the writer is struggling with basic comprehension [17, 18]. We hypothesized that students’ ability to write about data also might be negatively impacted when students struggled to comprehend the conceptual system they were asked to interrogate. However, we found no correlation between mean 4C scores and any assessment of conceptual material (data not shown). Nor was there an association between mean 4C scores on the lab reports and the related sections of the final (data not shown). Together, these data suggest that conceptual comprehension does not impact writing of a QC statement.

**Fig 5:**
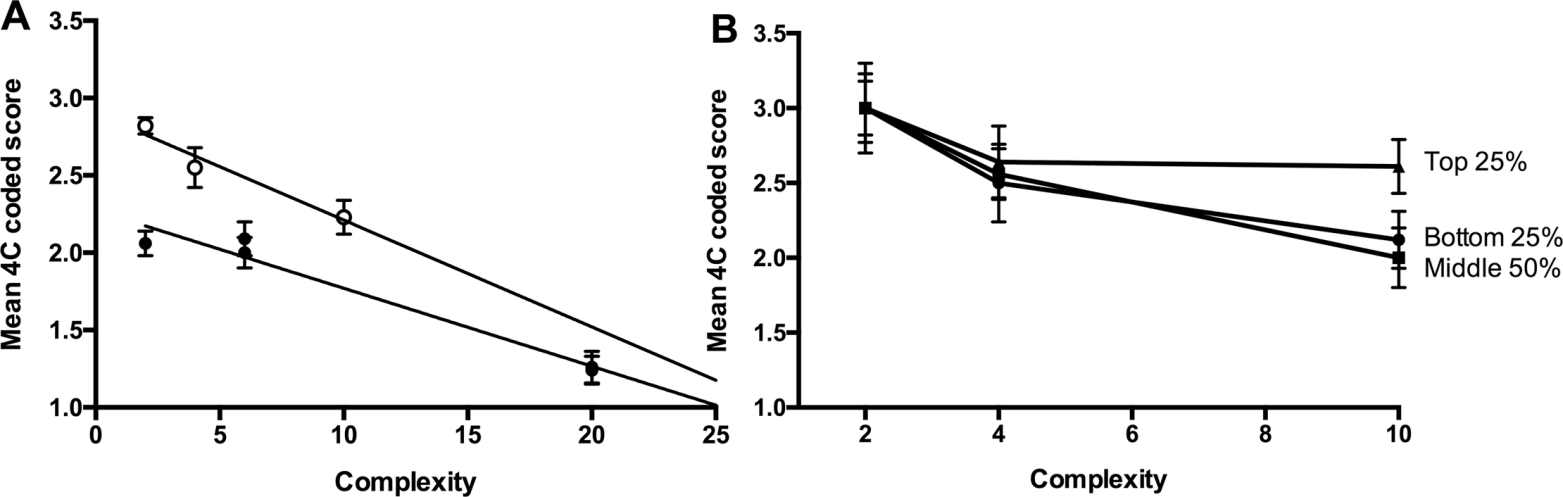
Writing syntax is negatively impacted by complexity but can be improved with writing support. (A) Writing syntax as a function of complexity measured by 4C scoring and reported as either unsupported (closed circles) or supported (open circles) by instructional intervention. Linear regression lines are shown (unsupported, R^2^ = 0.9644, supported R^2^ = 0.9471). (B) Students were stratified based on overall performance in the course. Statements from students within the group were averaged and reported. Error bars represent SEM.

In addition to conceptual understanding, QC statements require that the writer parse through the data set to select the relevant data points to interrogate. We hypothesized that the number of data points (values) in the data set may negatively impact QC statement syntax. We calculated the complexity of different assignments (see methods) and plotted mean 4C scores as a function of complexity index. We performed linear regression analysis on those mean 4C scores from writing samples occurring prior to formal writing intervention (2014 and 2015 lab reports, and the 2016 pre-test, Fig 5A, closed circles) and those that occur after specific writing intervention (2016 lab reports and 2016 post-test, Fig 5A, open circles). There is a strong inverse correlation between writing as measured by mean 4C scores and complexity (r^2^ = 0.9471 for supported and r^2^ = 0.9644 for unsupported writing, Fig 5A). Moreover, the slopes of the lines generated from the regression analysis of mean 4C scores do not vary significantly despite writing interventions (p = 0.3449). Although the task complexity in 2016 was reduced relative to 2015, the negative impact of complexity on writing persisted. Thus, as the complexity of experimental data sets increases, the ability to write clearly decreases regardless of the writing intervention.

### Complexity differentially impacts specific populations of students

Part of the developmental process of analytical reasoning is parsing relevant from irrelevant data [1]. We asked if subpopulations of our students were more capable of parsing information from larger data sets than others. We stratified 2016 students into quartiles based on overall performance in the course. We measured the mean 4C scores from the post-test and both lab reports, and plotted mean 4C score as a function of “constrained” complexity (Fig 5B). At lower complexity levels, there is no significant difference between the highest performing students and the lowest performing students (t test, p >0.05). Increasing complexity also had a negative impact on most of our students. However, students in the top quartile were less affected by increased complexity than the lower 75% of the class (t test, p <0.05, Fig 5B). These data suggest there are students who are developmentally capable of controlling the complexity of the task to focus on the skill of writing.

## Discussion

We set out to help STEM students write more clearly and we focused on writing a specific but universal form of evidence statement, the quantitative comparative statement ([14, 15], Fig 2). By analyzing text from student lab reports and professional scientific articles, we defined the syntax of quantitative comparative statements (Fig 1, Table 1). Based on the syntactic rules we established, we scored individual quantitative comparative statements and measured writing quality (Figs 3–5). Our data show that writing quality (measured by 4C scoring) can be improved with focused practice and feedback (Figs 3 and 4). Finally, our data show that the circumstance, i.e., the complexity of the writing task, influences writing quality. For example, writing quality decreased when students interrogated larger data sets (Figs 4 and 5), but was improved when students were directed by the writing prompt to focus on a subset of the data (Fig 5 and data not shown).

Our findings are consistent with previous research in Writing Studies and English Composition showing that syntax suffers when writers are confronted with complex and unfamiliar conceptual material [17, 18, 19]. The Cognitive Process Theory of Writing states that writing is a cognitive endeavor and that three main cognitive activities impact writing, the process of writing (syntax, grammar, spelling, organization, etc.), the task environment (the purpose of the writing task), and knowledge of the writing topic [17, 18, 19]. The theory posits that cognitive overload in any of these areas will negatively impact writing quality [17, 18]. Consistent with the theory, our data show that writing quality is a function of explicit writing practice (Fig 3), the size of the data set (Fig 4A compared to 4B) and scope of the writing prompts (Fig 4B 2015 compared to 2016).

### Explicit sentence level practice improves writing quality

Our data suggest that practicing isolated sentence construction improves writing quality (Figs 3 and 4). In every year of this study, we provided students with generalized feedback about their quantitative comparative statements (e.g., “needs quantitation” or “needs a comparison”) within the context of their lab report. In 2016, students practiced writing a QC statement related to their data but separate from the lab report. Although our feedback was the same, we observed improvement only when the feedback was given to QC statements practiced out of the lab report context (Fig 4A compared to 4B). Consistent with our data, the Cognitive Process Theory of Writing predicts that practicing specific syntax will increase fluency, lower the cognitive load on the writer’s working memory, and improve writing [17,18]. Our data are also consistent with research in English Composition demonstrating that when instructors support sentence-level syntax, they observe improved sentence level construction, improved overall composition, and higher level critical thinking [20]. In addition to improved sentence level syntax, we also observed overall quality of lab reports improved 12% in 2016 compared to the same lab report in 2015 (based on rubric scores, data not shown). If students develop a greater facility with the process of writing by practicing sentence level syntax, they have more cognitive resources available to develop and communicate their reasoning (our data, [20, 21]).

### Complexity of the writing task affects writing quality

We defined the complexity of the writing assignment as the landscape of information students must sample to interpret and communicate their data. In the case of lab reports, that information is the collected and analyzed data set (Table 2). Students interrogating a larger data set produced lower quality QC statements than when they interrogated a smaller data set (compare lab report #2 to lab report #1 in both 2014 and 2015 cohorts, Fig 4). In lab report #2, students not only contended with a larger number of values in the dataset compared to lab report #1, but also with two different measurements. These data are consistent with the Cognitive Process Theory of Writing that suggests that when demands on the writer’s knowledge of the topic increase, the writer cannot devote as many cognitive resources to the task environment or process of writing [17, 18]. However, we observed that the negative effect of experimental complexity on writing quality can be mitigated by writing prompts that focus students on a smaller, specific subset of the data (Fig 5A). More focused writing prompts and smaller data sets reduce the task environment of the assignment and allow more cognitive load to be devoted to the process of writing.

### Model for writing quality as a function of complexity

**Fig 6:**
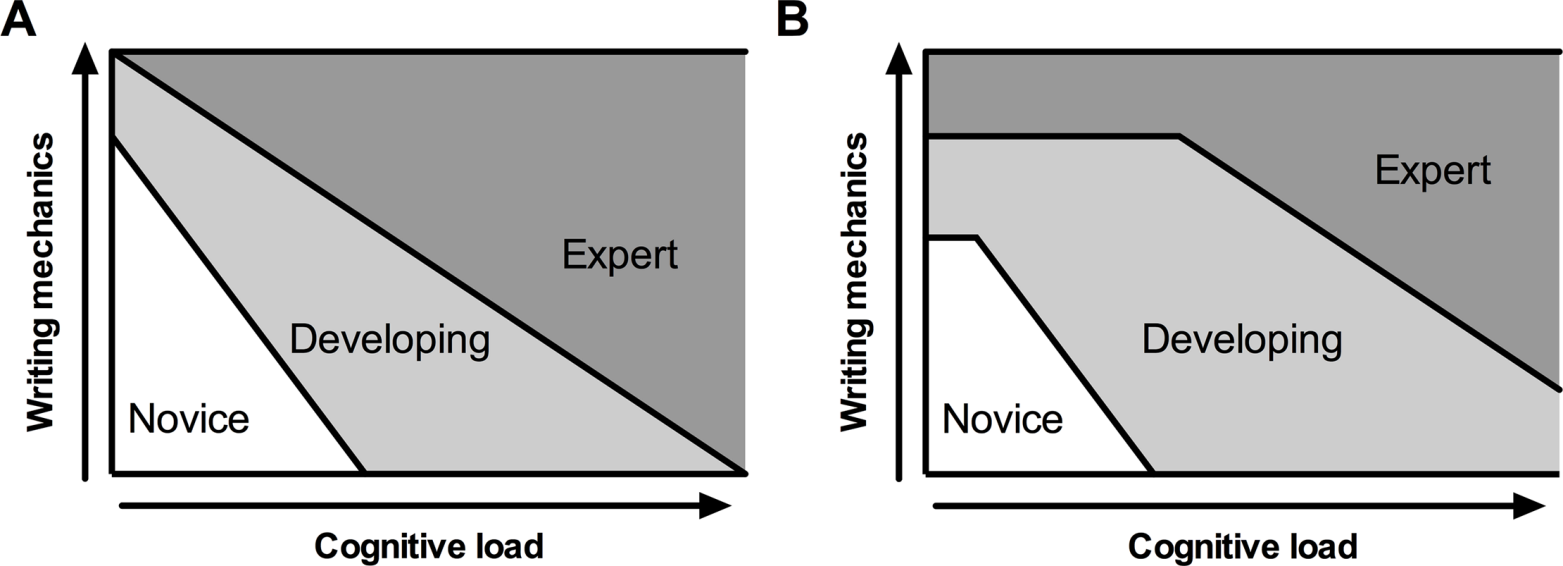
Model describing the effect of complexity on writing ability. (A) Simple linear model of the relationship between writing quality and complexity (cognitive load). (B) Model of the relationship between writing quality and complexity in which low complexity has minimal impact on writing quality but higher complexity negatively impacts writing quality.

Interestingly, the writing quality of students who finished the course with higher final grades (top quartile) was more resistant to increases in complexity compared to their classmates (Fig 5B). These data are consistent with the ideas of McCutchen who posits that as writers become more expert in their field, they have more cognitive resources to devote to clear communication. McCutchen suggests that expert writers have 1) more knowledge of their discipline, 2) more familiarity with the genres of science writing (task environment), and 3) more practice with the process of writing [19]. Based on research in Writing Studies, the Cognitive Process Theory of Writing, and the data presented here, we developed a predictive model of the impact of complexity (cognitive load) on writing quality (Fig 6). We have hypothesized a linear model in which any increase in complexity negatively impacts writing quality (Fig 6A) and a “breakpoint” model in which writers maintain a constant level of writing quality at lower complexity levels writing quality but decline at higher levels of complexity (Fig 6B). We hypothesize that our top performing students have moved into a more expert space in the model by developing strategies to parse a complex task environment and ignore irrelevant information. Effectively, these skills allow them to minimize the impact of complexity on their cognitive load and maintain their writing quality even in the face of complex data sets (Fig 5B).

### 4C instruction as a writing intervention

In addition to altering the writing assignment to decrease cognitive load on the students, we also think it will be important to provide students with syntactic structures at the sentence level. In this study, we did not use 4C annotation as an instructional intervention so that 4C scoring would be a more objective measure of writing quality. But, subsequent to this study, we and others have used 4C annotation as an instructional tool and found that student writing improves dramatically (data not shown). Although some argue that using overly structured or templated sentences can stifle creativity, providing basic structure does not necessarily lead to pedantic writing [22]. A commonly used text in college writing, “They say, I say,” determined that providing templates for constructing opinions and arguments gives students a greater ability to express their thoughts [23]. Specifically, weaker writers who lack intuitive understanding of how to employ these writing structures benefit from the use of explicit templates, while more advanced writers already employ these writing structures in a fluid and nuanced manner [23].

### 4C template as a foundation of quantitative writing

As students become more expert writers and write more complex and sophisticated sentences, they may choose to deviate from the proscribed sentence structure and make editorial decisions about the elements of the quantitative comparison in the context of their argument [23]. In fact, when we examined the 4C scores of quantitative comparative statements in published literature, we found that, on average, professional scientists write comparisons that are missing one of the three elements (4C score = 1.89 +/- 0.05, n = 281). The expert writer may eliminate an element of the evidence statement because he/she presumes a more sophisticated audience is capable of inferring the missing element from prior knowledge or within the context of the argument. Or, the author may provide all elements of quantitative comparison in their argument but not within a single sentence.

### Helping students become expert writers

Based on our research, we think novice writers should write for novice readers and include all of the syntactic elements of a QC statement. As students develop their professional voice, the 4C template will serve as a touchstone to frame their quantitative arguments, and the editorial choices they make will depend on the sophistication of their audience. Students will write clear arguments even if those elements no longer reside within the rigid structure of a single QC statement with a perfect 4C score. We are confident that by supporting student writing at the level of syntax, we are building a solid foundation that will give students greater capacity for reasoning in the face of increasing experimental complexity.

## Supporting information

S1 Fig Pre Test / Post Test. Example of the pre- and post-test used to assess the ability to interpret graphical and tabular data and write a quantitative comparative statement.
(PDF)

S2 Fig Lab Report Rubric. A detailed rubric provides students with explicit guidance for each lab report. This rubric corresponds with the experiment exploring enzyme kinetics of β-galactosidase.
(PDF)
