# Supplementary material for: Improving quantitative writing one sentence at a time
Source: PLoS One. 2018 Sep 12;13(9):e0203109. doi: 10.1371/journal.pone.0203109 (PMC6135501; doi:10.1371/journal.pone.0203109)
Supplement: S1 Fig — Example of the pre- and post-test used to assess the ability to interpret graphical and tabular data and write a quantitative comparative statement. (PDF) [file pone.0203109.s001.pdf]

Name________________________________
Lecture 9:15 10:30 1:00 (circle one)

Lab Section______________________

Look at the data presented in the graph and table below, then turn the page to answer some questions based on your interpretation of the data.

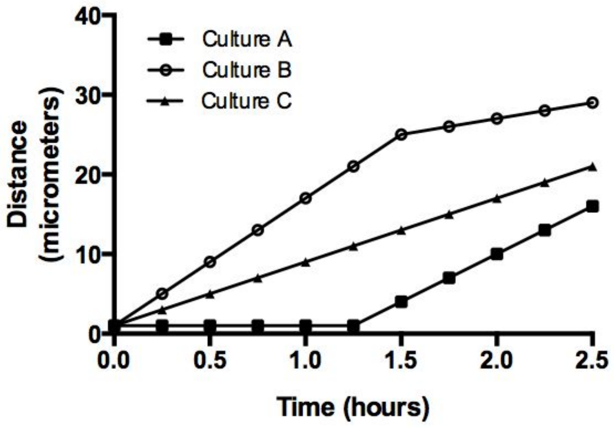

Figure 1. Movement of mammalian cells.

Table 1. Raw data of cell movement under three conditions.

Time (hours)	Culture A Distance (μm)	Culture B Distance (μm)	Culture C Distance (μm)
0	1	1	1
0.25	1	5	3
0.50	1	9	5
0.75	1	13	7
1.00	1	17	9
1.25	1	21	11
1.50	4	25	13
1.75	7	26	15
2.00	10	27	17
2.25	13	28	19
2.50	16	29	21

Use the graph and table to answer the following questions.

1. What is the shortest length of time the cells are moving (duration of movement) in any culture?

2. Calculate the fastest rate of movement for the cells in Culture B.

In each box, write a quantitative, comparative **sentence** that addresses the prompt. Support your conclusion with data from the graph, the table, and/or your answers above.

3. What is the difference in rate of movement between Cultures B and C from T = 0.5 to T = 1 hour?

4. How does the maximal rate of movement of Culture A compared to the maximal rate of movement in Culture B?

5. Compare the two slowest rates of movement in the last 45 minutes of the experiment.

Calculations here:
