# Supplementary material for: Improving quantitative writing one sentence at a time
Source: PLoS One. 2018 Sep 12;13(9):e0203109. doi: 10.1371/journal.pone.0203109 (PMC6135501; doi:10.1371/journal.pone.0203109)
Supplement: S2 Fig — A detailed rubric provides students with explicit guidance for each lab report. This rubric corresponds with the experiment exploring enzyme kinetics of β-galactosidase. (PDF) [file pone.0203109.s002.pdf]

This lab report will emphasize data presentation and data analysis. This report should be about 3 pages long! Edit your tables, graphs and text to find redundant and superfluous information and still retain the meaning of your ideas.

Download 2018 Enzyme Kinetics Class Data to complete your analysis.

Specific components of this analytical report: Turned in on Camino as a pdf.

Title your pdf file Lab section # Last Name First Initial EKreport (i.e., 2SabatierCEKreport.pdf).

Part 1 - Results.

Table 1: Average activities calculated from the class data collected in the Week 5 lab, include only class data for the enzyme preparations you will be comparing in the analysis section. Include the relevant average class values (± SEM).

Methods (optional) - If you excluded any part of the class data in your analysis, please include a statement describing the criteria you used to determine what data to include.

Figure 1

- Panel A: Michaelis-Menten Plot

- Panel B: Lineweaver-Burk Plot

For both graphs, include only class data for the enzyme preparations you will be comparing in your analysis (average ± SEM). Only one figure caption necessary for both of these figures.

Part 2 - Summary Table and Model

Table 2: Derived K_m and V_{max} values (average +/- SEM) of the class data for the enzyme preparations that are relevant to your analysis.

Figure 2: Model of β-galactosidase interacting with ONPG substrate summarizing the data in your analysis. These models should be consistent with your data. Feel free to use the model presented in lecture to craft your model figure. Include a figure caption.

Part 3 - Brief Analysis Section (0.5 page maximum)

Based on the class data, what effect do glucose and galactose have on β-galactosidase binding AND catalysis? Provide quantitative evidence for your argument using quantitative, comparative statements. Be explicit about what data you are comparing for

every statement you make. Provide statistical evidence when discussing significance of differences.

Part 4 - References (if necessary)

Include any references that contributed to your analysis in MLA format. In the text refer to the specific reference (first author's last name, year of publication) that is contributing to a specific section of text.

General instructions:

1. Be concise and precise.
2. Back up your statements with evidence or references [Cite references in text as (first author's last name, year of publication) and list the full MLA style reference at the end of your analysis].
3. Be quantitative. "A lot", "vanishingly small", "dramatically different" are not quantitative.
4. Be comparative when possible. *E.g.*, There is a 4-fold increase of this compared to that.
5. Write in active voice.
6. All tables and figures should be numbered and have titles (table) or captions (figures) detailing any information needed to interpret the data.

Rubric

Criteria	Ratings
TABLE 1: Table of average activities	5.0 pts<br>All data sets appropriate to discussion are included; Table conventions followed; No methods; Sample size and data exclusion criteria explicit.
FIGURE 1 - Michaelis-Menten and Lineweaver-Burk	5.0 pts<br>Include all class data (average +/- SEM); presentation of data is professional; trendline or connect the points as appropriate
FIGURE 1 title and caption	5.0 pts<br>Caption convention followed; legend included as necessary; trendline equations in caption or embedded in graph; no methods or analysis.
TABLE 2 - calculated Km and Vmax	5.0 pts<br>All data sets appropriate to discussion are included; method of calculation clear; values consistent with data presented; Units are included and correct; presentation is clear
FIGURE 2	5.0 pts<br>Figure is consistent with data presented. Figure title and caption follows convention, provides context for the figure and does not include methods or analysis.
ANALYSIS: Correct Evidence Statements	5.0 pts<br>Magnitude of effect is clear and consistent with statistical analysis; Comparisons are accurate; Calculations are consistent with comparisons; Consistent with data.
ANALYSIS: Evidence Statement Syntax	5.0 pts<br>Evidence statements are quantitative, comparative, and clear. Comparisons and context are explicit. Statistical analysis is referenced appropriately.
ANALYSIS: Effect of glucose and galactose on binding and catalysis	5.0 pts<br>Km or Vmax are referenced appropriately; terminology used appropriately; conclusions consistent with data. Calculations are accurate and consistent with statistical analysis.
OVERALL STYLE	5.0 pts<br>Sentences are complete; Writing is mostly in active voice; Very few typos; Appropriate vocabulary is used; Flow of arguments is logical; references are appropriate to point being made and in MLA format; appropriate figures are referenced.
